# Intramuscular desmoid tumor of the leg leading to external popliteal sciatic neuropathy: A case study and literature review

**DOI:** 10.1016/j.ijscr.2024.109333

**Published:** 2024-02-02

**Authors:** Ayoub Boushabi, Hicham Ait Benali, Mohammed Shimi

**Affiliations:** Orthopedics and Trauma-surgery Department, MOHAMMED VI University Hospital Center, Tangier, Morocco

**Keywords:** Desmoid tumor, Aggressive fibromatosis, Rare tumor, External popliteal sciatic neuropathy, Leg

## Abstract

**Introduction and importance:**

Desmoid tumors (DT), rare benign neoplasms of soft tissues, exhibit local aggressiveness and high recurrence rates. Originating from myofibroblast proliferation, complete surgical intervention is the preferred treatment. Despite their benign nature, these tumors are infrequent, predominantly affecting women between 15 and 60, with a higher incidence in adolescence.

**Case presentation:**

A 44-year-old woman with a DT in the leg mimicking external popliteal sciatic neuropathy. Diagnosis confirmed by biopsy, surgery performed with preservation of the external popliteal nerve, ensuring optimal nerve function. Two-year follow-up with no recurrence, demonstrating the success of the surgical intervention.

**Clinical discussion:**

DTs, although rare, exhibit three distinct genomic mutations, with the 45F genotype associated with the highest risk of recurrence. Generally sporadic, these tumors can be linked to familial adenomatous polyposis (FAP) and influenced by states of hyperestrogenism. DTs typically present as deep-seated masses, with frequent local recurrence despite complete resection.

**Conclusion:**

DTs pose diagnostic and therapeutic challenges, often requiring complete surgical intervention. Management depends on symptomatology, with careful monitoring for small asymptomatic tumors and adjuvant radiotherapy in case of incomplete resection. Despite surgical success, frequent recurrence underscores the need for in-depth research to enhance therapeutic approaches.

## Highlights

• Desmoid tumors, also known as aggressive fibromatoses, are rare and benign neoplasms of soft tissues, constituting less than 3% of soft tissue tumors.
• Female Predominance: These tumors primarily affect women and can manifest between the ages of 15 and 60, with a higher incidence in early adolescence, peaking around the age of 30.
• Rare Occurrence in the Leg: Although desmoid tumors can develop in various parts of the body, their manifestation in the leg is exceptionally rare, adding to the complexity of diagnosis and management.
• Therapeutic Challenges: Managing desmoid tumors poses a challenge. Despite complete resection, the risk of local recurrence remains significant, and there is no clear consensus on the most effective therapeutic approach. Chemoradiotherapy shows limited effectiveness.
• Illustrative Case of Mimicry: The study presents a case of a desmoid tumor in the leg mimicking an external popliteal sciatic neuropathy in a 44-year-old patient, highlighting the intricate diagnostic challenges associated with this exceptionally rare neoplastic entity.

## 1 Introduction

Desmoid tumors (DT) are rare and benign mesenchymal neoplasms, but they are characterized by their local aggressiveness [1]. They originate from the proliferation of myofibroblasts within a loose collagen matrix, starting in the connective tissues of fascia, aponeuroses, and intermuscular septa of striated muscles [2]. Despite their benign nature, these tumors account for less than 3 % of all soft tissue tumors. The exact pathogenesis of DTs remains unknown, although traumatic, endocrine, and genetic factors have been suggested as contributing elements.

The primary treatment for DTs is surgery; however, in cases of inoperable forms or recurrences, alternative therapeutic options have been considered to address this issue. These tumors, also referred to as locally aggressive fibromatoses, are notable for their high rate of local recurrence, even after extensive and complete resection [3]. We report the case of a patient with a desmoid tumor in the leg with compression of the external popliteal sciatic nerve, contributing to the understanding of this rare condition, and through which we will update our knowledge about this exceptionally rare neoplastic entity.

## 2 Case presentation

Our clinical case involves a 44-year-old female patient, a mother of two children, with no significant medical history, who presented for the management of a long-neglected mass on the right calf that had been evolving for over two years.

**Fig. 1:**
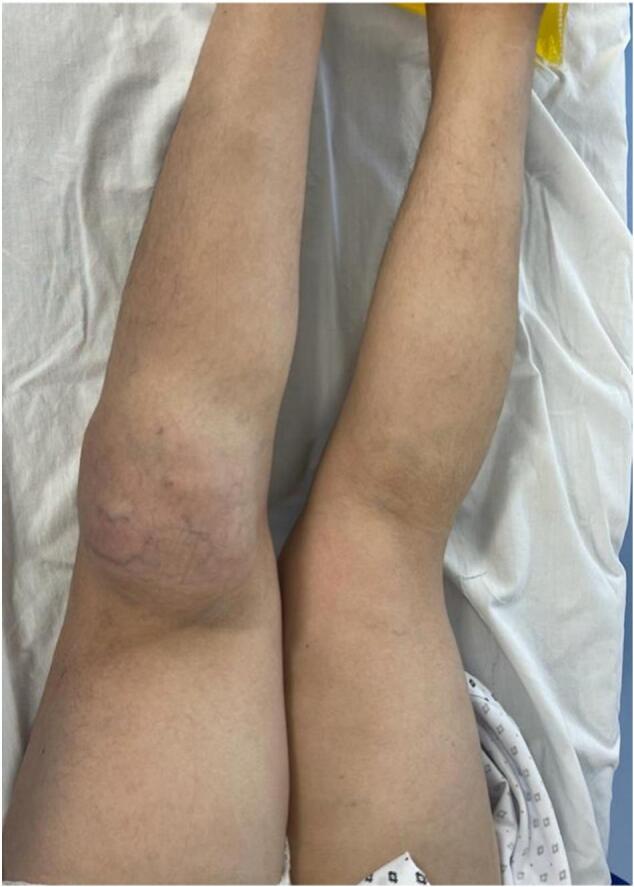
Clinical aspect of the tumor.

**Fig. 2:**
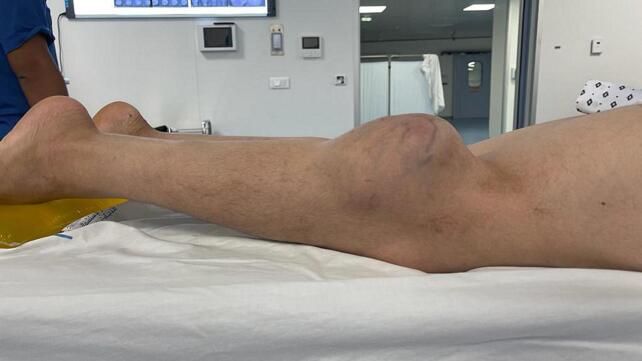
Clinical aspect of the tumor (calf mass).

On clinical examination, the mass was painful, tender, firm, and fixed in the deep plane, measuring 10 cm along its major axis, without inflammatory signs (Fig. 1) (Fig. 2), with the presence of collateral venous circulation. The examination also revealed a tingling sensation, muscle weakness in the toes, and a slight decrease in knee and ankle reflexes, indicative of compression of the external popliteal sciatic nerve. Lymph node areas were clear. The patient's general condition was preserved with no associated clinical signs.

**Fig. 3:**
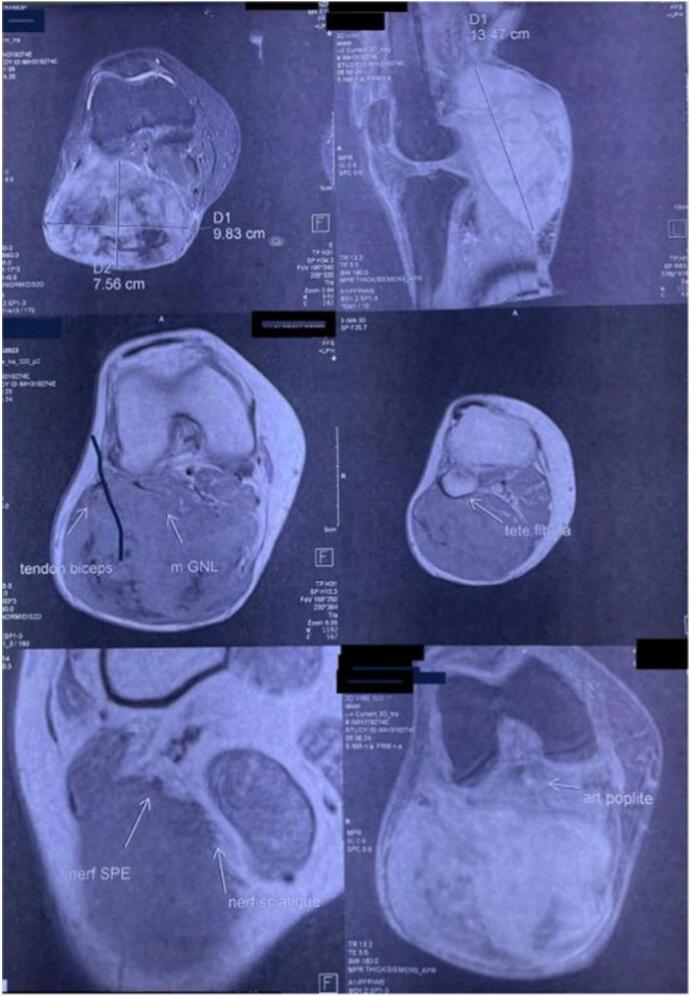
MRI image of the desmoid tumor.

**Fig. 4:**
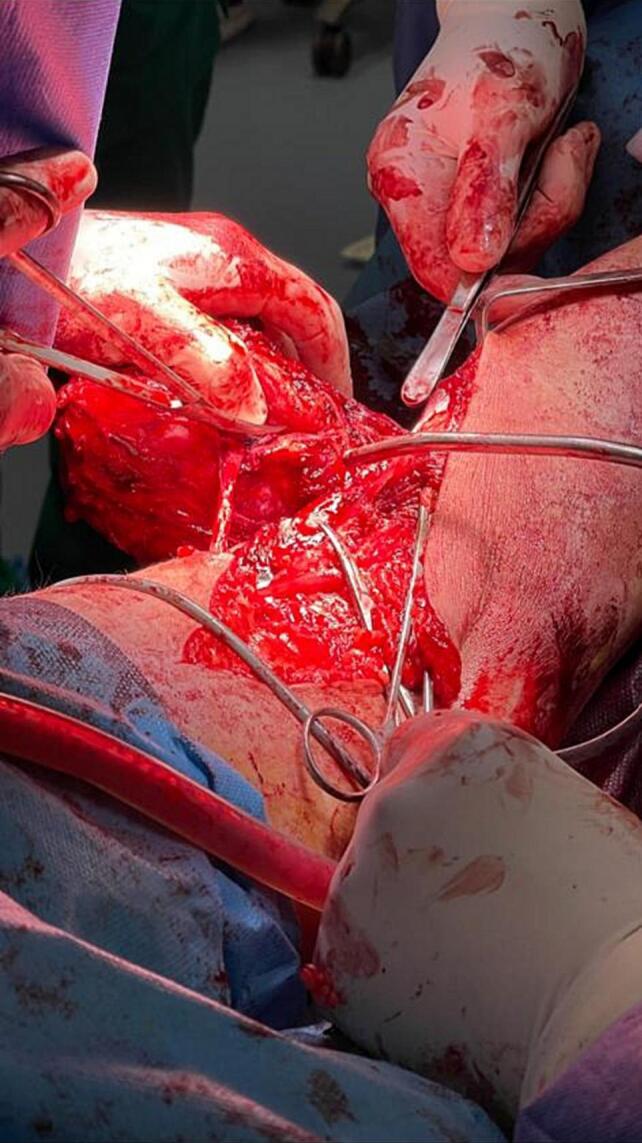
Intraoperative image showing the invasion of the external popliteal sciatic nerve by the desmoid tumor.

**Fig. 5:**
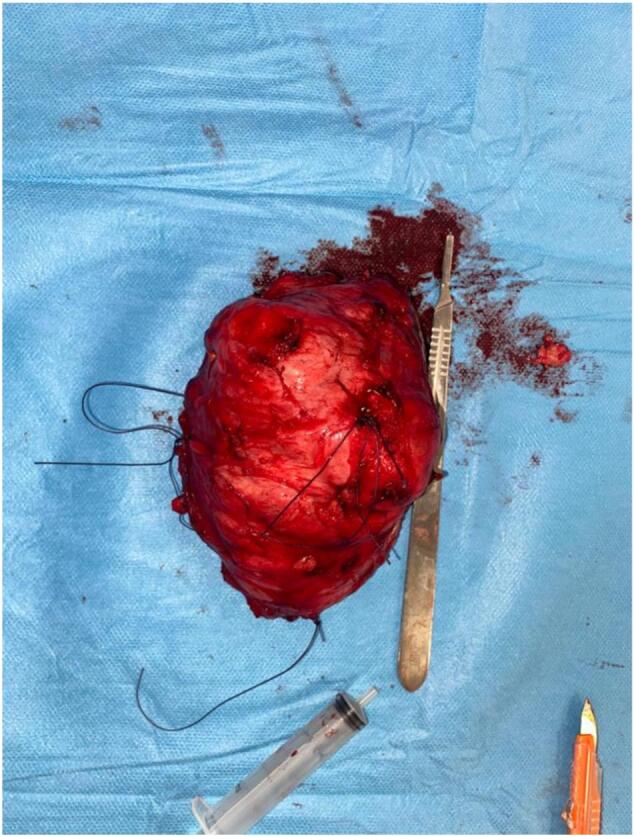
Postoperative specimen image after tumor resection.

The standard X-ray showed no particular findings. Magnetic resonance imaging (MRI) revealed an isointense mass in T1 and hyper-intense in T2 (Fig. 3). An initial biopsy was performed, confirming a desmoid tumor. During the surgical intervention, exploration revealed the invasion of the external popliteal sciatic nerve by the tumor mass, necessitating tumor resection while preserving the integrity of the nerve to minimize postoperative neurological complications. This approach successfully removed the tumor while maintaining nerve function, ensuring a better functional prognosis for the patient (Fig. 4). The surgical specimen underwent histopathological examination, conclusively confirming the diagnosis of a desmoid tumor (Fig. 5).

After a two-year follow-up, the patient's progress has been very favorable, marked by the absence of desmoid tumor recurrence. The patient has regained optimal function of her external popliteal sciatic nerve, demonstrating the success of the surgical intervention.

## 3 Dicscussion

Although desmoid tumors are extremely rare, most of these neoplasms occur sporadically. Three distinct genomic mutations have been identified in the context of these tumors, namely 41A, 45F, and 45. Notably, the 45F genotype is associated with the highest risk of recurrence. Traditionally, desmoid tumors are associated with familial adenomatous polyposis (FAP), primarily in its abdominal form resulting from a mutation in the APC gene. It is worth noting that desmoid tumors can be influenced by states of hyperestrogenism, such as pregnancy. Despite their rarity, they constitute less than 3 % of all soft tissue neoplasms, although their prevalence can reach up to 13 % in patients with colonic FAP. Generally, these tumors affect women more than men and most commonly occur between the ages of 15 and 50 [[4], [5], [6]].

From a histological perspective, desmoid tumors are characterized by the presence of small bundles of spindle cells within an abundant fibrous stroma. It is crucial to note that these spindle cells are actually monoclonal fibroblasts proliferating. The tumor typically exhibits low cellularity, and the cells do not display any nuclear or cytoplasmic features of malignancy [4,5]. In most cases, desmoid tumors present as slow-growing, deep-seated masses that are typically painless or minimally painful. Common sites of clinical presentation include the abdominal wall, trunk, and rarely the extremities. Multifocal presentation is rare. Interestingly, one in four patients reports a history of trauma or surgery at the site of tumor development. Tumor recurrence can occur locally or regionally but never at a distant site from the initial tumor (non-metastatic). Computed tomography and MRI are recommended for diagnosis and monitoring of these tumors. On T1-weighted images, desmoid tumors appear hypo- or isointense compared to muscles, while on T2-weighted images, they appear hyperintense. With gadolinium contrast, desmoid tumors show moderate enhancement with hypointense bands reflecting collagen bundles.

Post-surgery follow-up is essential to assess the effectiveness of the intervention and detect any early recurrence. Radiological confirmation of suspicion should always be supported by a biopsy of the tumor. The definitive diagnosis is established by performing a biopsy of the tumor. Biopsy indications include diagnostic confirmation before surgery; however, they must be carefully evaluated due to the potential risk of stimulating tumor growth. Therefore, its use should be restricted to situations where the differential diagnosis with a possible lymph node or carcinoma cannot be specified non-invasively.

Post-neoadjuvant treatment surgery should be tailored based on tumor response, and the surgical strategy should aim for maximal excision while preserving vital structures. Postoperative follow-up should be regular, with special attention to signs of recurrence and potential complications.

The treatment of desmoid tumors can be broadly categorized into three groups: asymptomatic resectable tumors, symptomatic resectable tumors, and unresectable and recurrent tumors. For the first category (asymptomatic resectable tumors), the literature predominantly suggests that a “watch and wait” approach may be a valid option for small tumors in regions not likely to impact function. If the tumor progresses, definitive therapy in the form of surgery or radiotherapy may be considered [[7], [8], [9], [10]]. For the second category (symptomatic resectable tumors), treatment is primarily based on surgery. The risk of recurrence depends on factors such as tumor size, location, patient age, and the margin of tumor resection. It is important to note that extra-abdominal tumors have a higher rate of recurrence than abdominal tumors. Recent studies have also shown that adjuvant radiotherapy may reduce the risk of recurrence after incomplete surgical resection, especially in patients with recurrent tumors [7]. For the third category (unresectable tumors), therapeutic options include systemic chemotherapy, radiotherapy, and close observation. Radical resection, such as amputation, should be avoided whenever possible. The response to radiotherapy in these cases may be slow but comes with a good rate of local control, reaching up to 81.5 % [10] [11].

## 4 Conclusion

Desmoid tumors represent a complex diagnostic rarity, eliciting a lack of consensus regarding their therapeutic management. Their limited sensitivity to chemo-radiotherapy underscores the importance of surgery as the sole curative treatment for symptomatic cases. However, the high recurrence rate and the reserved prognosis for most patients emphasize the need for more in-depth research to enhance our understanding and therapeutic approaches to this rare condition. The exploration of new treatment and follow-up strategies should be encouraged to provide patients with desmoid tumors with an improved quality of life and better long-term outcomes.

## 5 Methods

This work has been reported in line with the SCARE 2023 criteria.

## Consent

Written informed consent was obtained from the patient for publication of this case report and accompanying images. A copy of the written consent is available for review by the Editor-in-Chief of this journal on request.

## Ethical approval

The ethical committee approval was not required given the article type (case report). Ethical Approval: Not applicable.

## Funding

This research did not receive any specific grant from funding agencies in the public, commercial, or not-for-profit sectors.

## Author contribution

BOUSHABI Ayoub: study concept, Data collection; data analysis; writing review & editing.

AIT BENALI Hicham: Contributor, Supervision and data validation.

SHIMI Mohammed: supervision and data validation.

## Guarantor

BOUSHABI Ayoub.

AITBENALI Hicham.

SHIMI Mohammed.

## Research registration number

As this manuscript was a case report with no new medical device nor surgical techniques, not prior registration is required.

## Conflict of interest statement

The authors state that they have no conflicts of interest for this report.
